# Tunable synthetic extracellular matrices to investigate breast cancer response to biophysical and biochemical cues

**DOI:** 10.1063/1.5064596

**Published:** 2019-02-08

**Authors:** Lisa A. Sawicki, Elisa M. Ovadia, Lina Pradhan, Julie E. Cowart, Karen E. Ross, Cathy H. Wu, April M. Kloxin

**Affiliations:** 1 Department of Chemical and Biomolecular Engineering, University of Delaware, Newark, Delaware 19716, USA; 2 Center for Bioinformatics and Computational Biology, University of Delaware, Newark, Delaware 19711, USA; 3 Department of Biochemistry and Molecular and Cellular Biology, Georgetown University Medical Center, Washington, DC 20057, USA; 4 Department of Materials Science and Engineering, University of Delaware, Newark, Delaware 19716, USA

## Abstract

The extracellular matrix (ECM) is thought to play a critical role in the progression of breast cancer. In this work, we have designed a photopolymerizable, biomimetic synthetic matrix for the controlled, 3D culture of breast cancer cells and, in combination with imaging and bioinformatics tools, utilized this system to investigate the breast cancer cell response to different matrix cues. Specifically, hydrogel-based matrices of different densities and modified with receptor-binding peptides derived from ECM proteins [fibronectin/vitronectin (RGDS), collagen (GFOGER), and laminin (IKVAV)] were synthesized to mimic key aspects of the ECM of different soft tissue sites. To assess the breast cancer cell response, the morphology and growth of breast cancer cells (MDA-MB-231 and T47D) were monitored in three dimensions over time, and differences in their transcriptome were assayed using next generation sequencing. We observed increased growth in response to GFOGER and RGDS, whether individually or in combination with IKVAV, where binding of integrin β1 was key. Importantly, in matrices with GFOGER, increased growth was observed with increasing matrix density for MDA-MB-231s. Further, transcriptomic analyses revealed increased gene expression and enrichment of biological processes associated with cell-matrix interactions, proliferation, and motility in matrices rich in GFOGER relative to IKVAV. In sum, a new approach for investigating breast cancer cell-matrix interactions was established with insights into how microenvironments rich in collagen promote breast cancer growth, a hallmark of disease progression *in vivo*, with opportunities for future investigations that harness the multidimensional property control afforded by this photopolymerizable system.

## INTRODUCTION

Breast cancer is the most common cancer diagnosed and one of the leading causes of cancer-related deaths in women worldwide.^1^ The extracellular matrix (ECM) surrounding breast cancer cells is thought to play a key role in tumor growth, metastasis, and survival at metastatic sites, providing structural support and biochemical factors that promote adhesion and signal transduction.^2,3^ For example, the tumor stroma undergoes changes throughout tumor development and progression, including degradation, redeposition, and crosslinking of collagens, with variations in matrix stiffness and composition which drive cell activation and migration.^4^ Similar tissue remodeling processes influence invasion and the growth or dormancy of disseminated tumor cells at metastatic sites.^5^ To understand critical cell-ECM interactions involved in these complex processes, *in vitro* model systems that capture key aspects of these tissue microenvironments, from native breast tissue to metastatic tissue sites, are needed for hypothesis testing.

Primary and metastatic tissue sites have distinct properties due to their different functions in the body.^6–8^ The ECM of these tissues provides a three-dimensional (3D) mechanical support for cells, consisting of insoluble proteins (e.g., collagen, laminin, fibronectin, and elastin), glycosaminoglycans (e.g., hyaluronic acid), and proteoglycans (e.g., aggrecan) that form a natural polymer network with different mechanical properties based on the tissue type and composition.^9,10^ Young's modulus (E), as a measure of matrix “stiffness”, has been reported for primary breast and metastatic tissue sites, ranging from “soft” (mammary tissue or organoids E ∼ 100–700+ Pa; bone marrow, E ∼ 600 Pa; liver, E ∼ 640 Pa) to “stiff” (breast tumors E ∼ 3000–5000+ Pa; lung tissue, E ∼ 2000–6000 Pa).^11–15^ As noted above, the stiffness and structure of ECM have been implicated as important factors in cell proliferation and motility in both tumor growth and metastasis, where cells exert traction forces on structural ECM proteins and degrade the local matrix to proliferate and ultimately leave the primary tumor or enter a metastatic site.^4,16^ Beyond the structure, insoluble ECM proteins also provide binding sites that allow adhesion to the matrix, which have been shown to promote cancer progression through binding cellular integrins, particularly β1 and αvβ3.^17^ Identification of critical mechanical and biochemical cues that regulate cell responses within this complex milieu is needed for a better understanding of the mechanisms regulating cancer progression and improving treatment strategies (e.g., therapeutic target identification and drug screening).

Different 3D *in vitro* culture models, both naturally derived and synthetic material-based systems, which capture aspects of the native tissue structure and composition have been developed to study cell-ECM interactions involved in cancer, as well as various processes related to disease, aging, and tissue repair. Naturally derived materials, including collagen matrices,^18^ basement membrane extract (BME),^19^ gelatin-methacrylate (gelMA),^20^ hyaluronic acid-based hydrogels,^21^ cell-secreted matrices,^22^ and combinations thereof,^23^ have been widely used due to their inherent bioactivity, providing a structure and sites for receptor binding and enzymatic degradation which promote cell viability and functions. In particular, BME or Matrigel, derived from Engelbreth-Holm-Swarm tumors and containing a variety of proteins (e.g., Laminin, Collagen IV, and Nidogen), proteoglycans (e.g., heparan sulfate), and other factors (e.g., growth factors and proteases), mimics aspects of the basement membrane found in epithelial and endothelial tissues and has been widely used.^24,25^ For example, in a seminal study, Bissell and coworkers reported how a large panel of breast cancer cells cultured in three dimensions within Matrigel adopted distinct morphologies and gene expression profiles reminiscent of their behaviors *in vivo* and distinctly different from observations in 2D cultures, revealing the importance of the microenvironment and dimensionality in regulating the responses of breast cancer cells *in vitro*.^26^ However, with such naturally derived materials, batch-to-batch variability and limited control of mechanical properties and biochemical content can make it challenging to test hypotheses about the role of specific ECM cues in cellular responses.^27,28^

Synthetic scaffolds, particularly hydrogel-based materials, have gained increasing interest in recent years for the culture of cancer cells *in vitro* owing to their ease of property control for mimicking aspects of different soft tissues. The formation of tumor spheroids has been reported in several polymer-based synthetic matrices, and behavior related to metastasis and response to drug treatments match that observed *in vivo*.^29–31^ For example, in early studies, Loessner *et al.* described the encapsulation of epithelial ovarian cancer cells within a poly(ethylene glycol) (PEG)-based hydrogel with tunable chemical and mechanical properties.^31^ Increasing matrix stiffness was observed to decrease the spheroid size, and the incorporation of an integrin-binding peptide sequence, RGD, increased cell proliferation within the system. In a complementary PEG-based hydrogel system, Gill *et al.* demonstrated the formation of lumenized lung adenocarcinoma spheroids in response to stiff matrices and higher concentrations of the adhesive RGDS peptide binding sequence.^29^ Specifically, in the study of breast cancer, such synthetic hydrogel-based materials have also been used to study spheroid growth amongst other cellular responses: these investigations further support the importance of multidimensional culture for observations of *in vivo*-like phenotypic characteristics, including the cell morphology, migration, cytokine secretion, and drug responses.^32–36^ New chemistries and processing have also been integrated to provide additional handles for synthetic matrix property control and high throughput evaluation of cell responses.^37–42^ Despite these advancements, heterogeneous responses of breast cancer cells often have been observed within individual samples for many of these systems, partly owing to variance in cell-cell and cell-matrix interactions within the sample and the gradients that develop as the cell number increases.^43,44^ Additionally, many synthetic culture systems do not fully capture key *in vivo* characteristics of progression; for example, increased proliferation and growth increased with matrix density and stiffness which occur natively with disease progression.^45,46^ Further, quantitative observations of cell morphology and cluster growth between different breast cancer cell subtypes are limited, partly owing to the significant differences in subtype responses to matrix properties that make direct comparisons challenging (e.g., where characteristics of a mesenchymal phenotype are exhibited by basal breast cancer cells and an epithelial phenotype exhibited by luminal).^40^ New approaches are needed to address these needs and examine quantitatively how combinations of insoluble matrix cues influence the activities of different types of breast cancer cells, from the cellular level (e.g., cell morphology and growth) to the gene level (e.g., transcriptome).

**FIG. 1.**
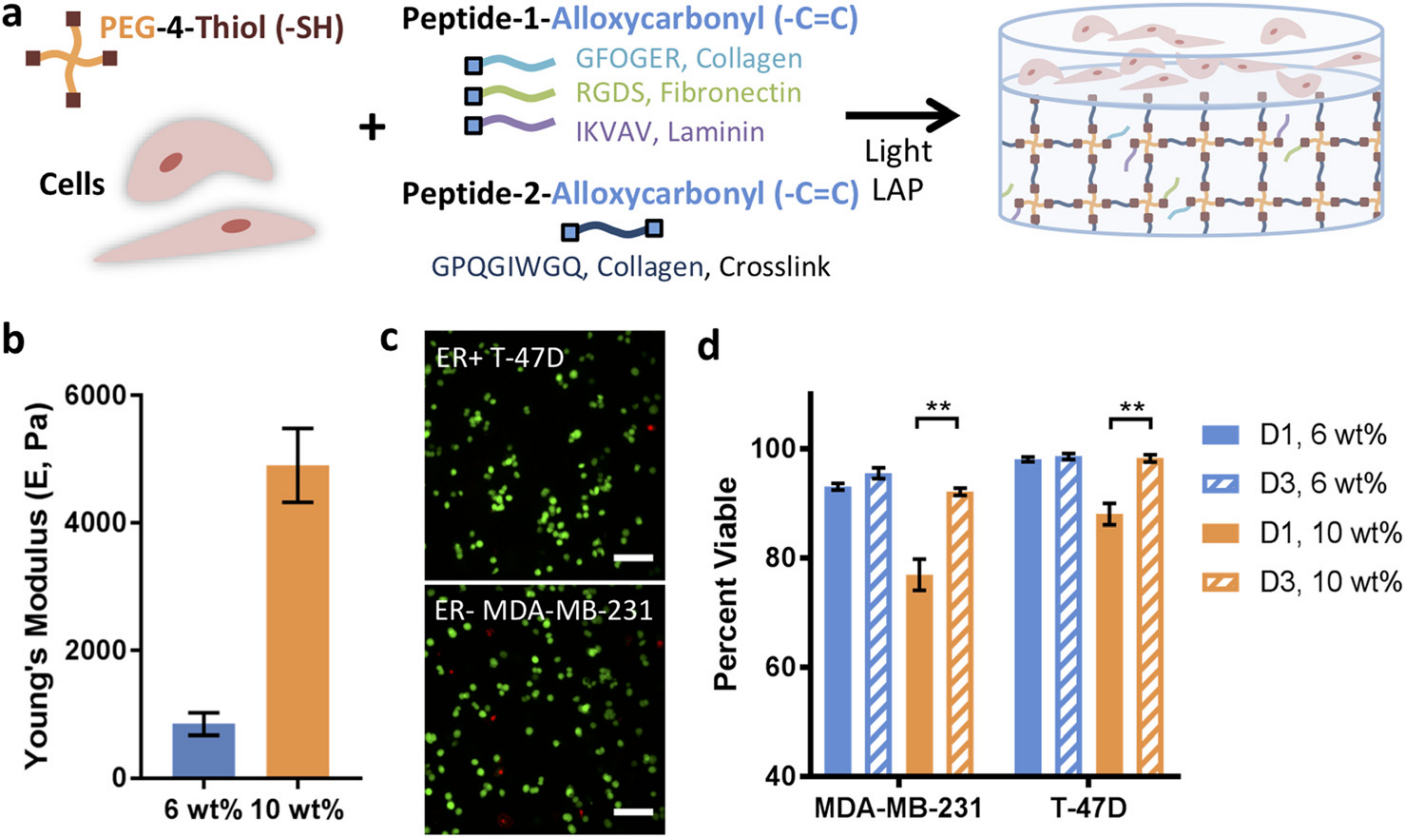
Synthetic matrices for breast cancer cell culture and encapsulation viability. (a) Well-defined hydrogel-based synthetic extracellular matrices were synthesized using a multiarm poly(ethylene glycol) tetra thiol (PEG-4SH), cell-degradable peptide crosslinks derived from collagen, and different integrin-binding pendant peptides derived from collagen, laminin, and fibronectin. Homogeneous, single cell suspensions of prototypical breast cancer cells from different breast cancer subtypes [basal MDA-MB-231 (ER−) and luminal A T-47D (ER+)] were encapsulated within the matrix during hydrogel formation. To allow facile handling and homogenous cell responses over time for quantitative image analysis, a thick cell-free base layer of hydrogel (15 *μ*l) was first formed, and subsequently, a thin layer of precursor solution containing cells (5 *μ*l) was polymerized on top. (b) The mechanical properties of these synthetic matrices were tuned to mimic different soft tissue microenvironments: here, a 6 wt. %–10 wt. % precursor solution was used to achieve moduli in the range of healthy, laminin-rich mammary epithelium (Young's modulus [E] ∼ 0.5 kPa) to collagen-rich tumors (E ∼ 5 kPa). (c) Good viability was observed for both ER− and ER+ breast cancer cells encapsulated within all matrix compositions. Representative images shown for day 1 (D1) 6 wt. % conditions (Z-stack projection, scale bar = 100 *μ*m) with (d) quantification of percent viable cells for 6 and 10 wt. % matrices at D1 and day 3 (D3). The data shown illustrate the mean (n = 3 samples per condition; >100 cells/sample counted) with error bars showing the standard error and statistical differences determined using Student's t-test (**p < 0.01).

Herein, we combine the high degree of user-directed property control afforded by photopolymerizable synthetic matrices with quantitative imaging and bioinformatics techniques for interrogating breast cancer cell responses to different matrix cues. Specifically, we establish an approach for the 3D culture of different breast cancer cell subtypes in well-defined, biomimetic synthetic matrices and implement a 3D imaging and bioinformatics workflow to investigate cell responses to the presentation of key biochemical and biophysical factors found within the tumor microenvironment (Fig. 1). We previously developed a method for the formation of hydrogel-based synthetic matrices with light-triggered thiol-ene chemistry using a bioinert multiarm poly(ethylene glycol) (PEG) functionalized with thiols and biomimetic peptides functionalized with alkenes (MMP-cleavable crosslinker and integrin-binding RGDS) and have established its utility for stem cell culture: this base matrix utilizes accessible monomers to facilitate use across fields and affords facile assessment of peptide incorporation using a colorimetric assay.^47,48^ Building upon this, in this contribution, we aimed to mimic aspects of the tumor microenvironment by varying matrix density to achieve relevant “soft” and “stiff” moduli and incorporating different biomimetic integrin-binding peptides, specifically those derived from key ECM proteins collagen [(POG)_3_PO**GFOGER**(POG)_4_], fibronectin/vitronectin (**RGDS**), and laminin (**IKVAV**). We hypothesized that a synthetic matrix rich in integrin-binding peptides derived from collagen and fibronectin/vitronectin, mimicking aspects of the remodeled epithelium that is observed natively during tumor progression, would activate breast cancer cells relative to a matrix rich in integrin-binding peptides derived from laminin, mimicking aspects of a healthy mammary epithelium, with the potential for synergies with matrix stiffness. To test this, less aggressive luminal A breast cancer cells (estrogen receptor positive [ER+] T47D) and more aggressive basal breast cancer cells (ER− MDA-MB-231) were selected as prototypical examples of breast cancer cells from different subtypes that exhibit different phenotypic characteristics (epithelial vs. mesenchymal), and these cells were encapsulated within discrete environments to examine their response to individual and combinations of matrix cues. Imaging-based methods for analysis of the cell and cluster morphology and size in three dimensions were established and utilized to quantitatively assess the response at the cellular level, where binding of β1 integrin was observed to be important in regulating activation (spreading and proliferation for ER− MDA-MB-231 and spheroidal growth for ER+ T47D). Further, we utilized next generation sequencing, bioinformatics analyses, and quantitative Reverse Transcription Polymerase Chain Reaction (qRT-PCR) analyses to probe the cell response to selected matrix compositions for broader insights. This work demonstrates a new approach for investigating breast cancer cell-ECM interactions with insights into how different combinations of biochemical cues within “soft” and “stiff” microenvironments promote breast cancer growth with broad relevance for investigations of breast cancer progression.

## RESULTS

### Cancer cells remain viable in synthetic matrices post-encapsulation

Hydrogels of different matrix densities and moduli were generated to mimic the properties of soft tissue ECMs [Fig. 1(a)], including aspects of a stiffer, collagen-rich matrix found natively during disease progression (Young's modulus [E] ∼ 5 kPa) and softer, laminin-rich epithelium found in healthy mammary tissue (E ∼ 0.5 kPa).^4^ Specifically, we selected low and high density matrices (6 and 10 wt. % with respect to PEG4SH) that rapidly formed with low, cytocompatible doses of light (Fig. S1) and were modified with different integrin-binding peptides to mimic key proteins within native tissue ECMs [RGDS = Fibronectin mimic, αvβ3 (strongest), α5β1, and others;^49,50^ GFOGER = Collagen mimic, α1β1, α2β1;^51^ IKVAV = Laminin mimic and laminin receptor^52^]. Moduli of the resulting hydrogels were measured after equilibrium swelling using rheometry, and storage moduli (G′) were converted to E using rubber elasticity theory for comparison to the reported values for different tissues. For 6 and 10 wt. %, matrix moduli of E = 850 ± 180 Pa and E = 4900 ± 580 Pa were observed [Fig. 1(b), no peptide control with 2 mM free thiol at preparation], respectively. Significant differences were similarly observed for 6 vs. 10 wt. % conditions for each pendant peptide composition (RGDS, IKVAV, or GFOGER) (Fig. S1). Importantly, no statistical difference was observed between no peptide and individual pendant peptides for 6 wt. % and 10 wt. % matrices, respectively. Further, within error, the 6 and 10 wt. % conditions have similar magnitudes to the reported values for healthy mammary and tumor tissues (E ∼ 100–700+ Pa and E ∼ 3000–5000+ Pa, respectively).^11–15^ Consistent incorporation of each peptide across compositions was verified using a previously published technique (Fig. S2).

In our preliminary experiments with these matrix compositions, we evaluated the use of hydrogel geometries that we and others had previously used for stem cell culture, where cells were uniformly encapsulated and cultured within a thicker hydrogel construct (20 *μ*l hydrogels with a thickness of ∼1.5 mm and a diameter of ∼5 mm).^47^ However, breast cancer cells encapsulated within these thicker samples exhibited an inhomogeneous cell response in the z-direction over time, with more cells and larger cell clusters observed near the top of the hydrogel by day 10 in 3D culture (Fig. S3). We hypothesized that decreasing the thickness of the cell-laden hydrogel and keeping it off the bottom of the culture plate toward facilitating mass transfer would lead to a more homogeneous cell response through the full thickness of the hydrogel. However, handling thin hydrogels over long culture times can be challenging (e.g., damage to hydrogels during media changes, transfer between plates, or processing for immunostaining). To address this, we formed layered hydrogels [schematic Fig. 1(a)]: a thicker bottom layer (15 *μ*l) was formed that is cell-free for ease of handling, and a thinner top layer (5 *μ*l, ∼500 *μ*m thick after equilibrium swelling) containing encapsulated cells was formed on top of it for quantitatively observing a homogenous cell response. The resulting constructs were robust, building upon seminal work by Bryant and coworkers, amongst others forming layered hydrogels with similar photopolymerized thiol-ene chemistry,^53^ and a uniform response of cells was observed throughout the cell-laden matrix (Fig. S3).

To initially evaluate the utility of this system for breast cancer cell culture, the viability of ER− MDA-MB-231 and ER+ T47D cells encapsulated within 6 and 10 wt. % hydrogel matrices was monitored post-encapsulation. At 1 and 3 days after encapsulation, cells were stained using a live-dead cytotoxicity assay, and the percentage of viable cells was quantified, where live cells with intact cell membranes stain green (Calcein-AM) and dead or dying cells with cell membrane damage stain red (Ethidium Homodimer) [Fig. 1(c)]. Cells remained viable in all matrix densities (>70%). Higher viability (>90%) was observed in the 6 wt. % matrix at day 1 and in both matrices at day 3 relative to the 10 wt. % matrix at day 1 (supplementary material Table 1). While the viability in 10 wt. % matrices was lower on day 1, perhaps owing to the increased confinement of the cells at this early time in culture, viability was greater than 90% for all matrix densities by day 3, indicating the survival of the cells that were viable directly after encapsulation. Notably, basal MDA-MB-231 cells expressed the mesenchymal phenotype marker vimentin, whereas luminal A T47D cells expressed the epithelial phenotype marker E-cadherin in these 3D cultures, with stellate and mast morphologies, respectively, reminiscent of cell responses observed in naturally derived BME matrices (Fig. S5).^26^ Additionally, to confirm that the culture system is broadly useful for 3D culture of different breast cancer cell subtypes, two other representative breast cancer cell lines were encapsulated (luminal A ER+ ZR-75‐1 and luminal B ER+ HER2+ BT474), where high viability was again observed (Fig. S6).

### Matrix density affects spreading and growth of breast cancer cells

Previously, increased matrix density has been shown to inhibit spheroid growth of lung adenocarcinoma^29^ and epithelial ovarian cancer cells.^31^ Additionally, in a PEG-Matrigel hybrid matrix, growth of normal and malignant mammary epithelial organoids was observed to be restricted in more rigid, dense microenvironments.^41^ Here, exploiting the flexibility of the photopolymerized thiol-ene synthetic matrix, we investigated the individual and subsequently combinatorial effects of the matrix density and biochemical content on different breast cancer cell subtypes.

**FIG. 2.**
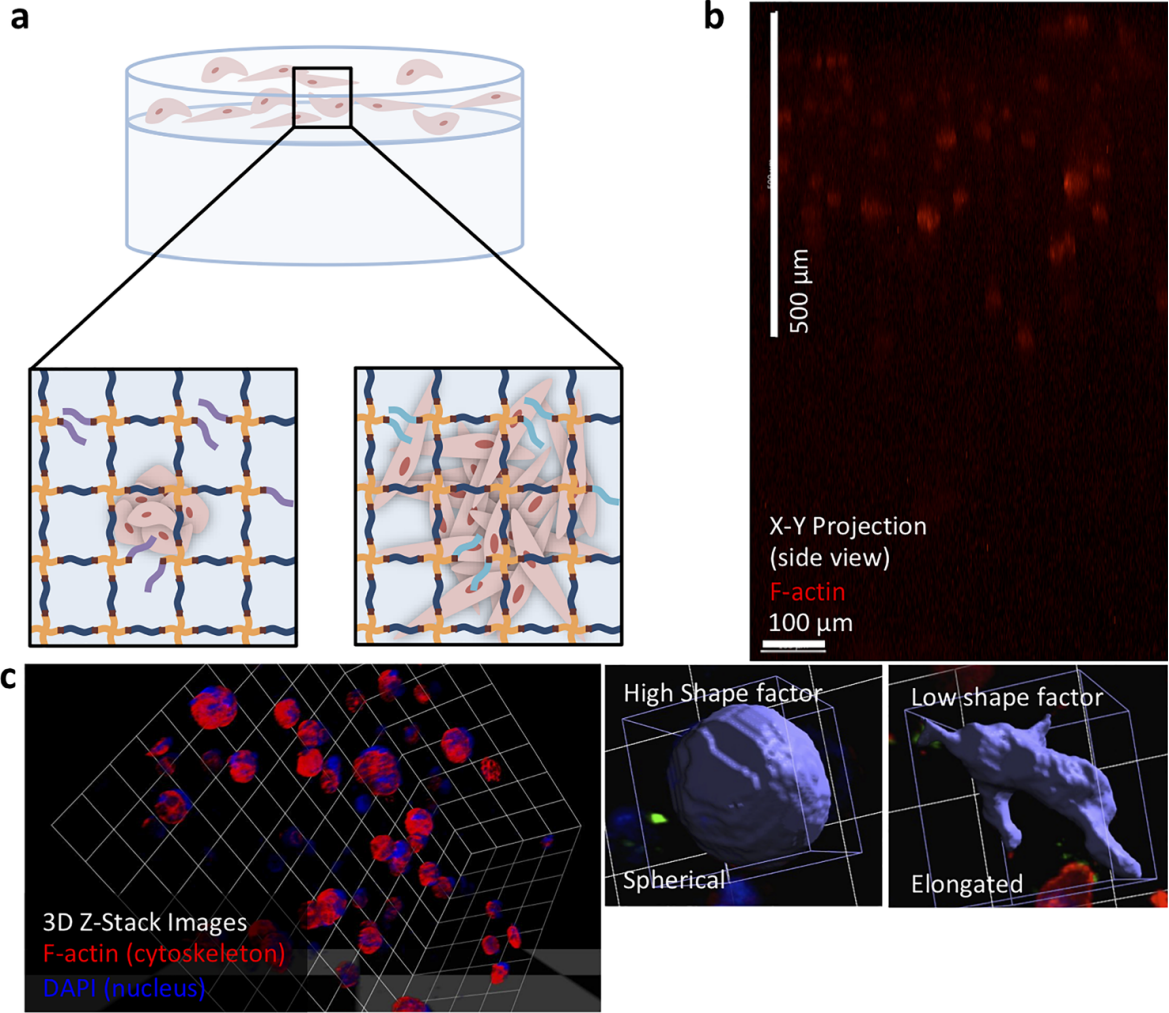
Schematic representation of the layered hydrogel approach and image analysis for assessing the cell response. (a) Cells cultured within synthetic matrices grow to form small spheroids (mass morphology) or elongated cells and clusters (stellate morphology) in response to different biochemical and biophysical cues over 10 days in culture. (b) Side view of confocal z-stack (1000 *μ*m scan) of the layered hydrogel cultures showed clear confinement of encapsulated cells within the top layer (500 *μ*m) and a cell-free bottom layer over time (here, MDA-MB-231 cells cultured for 10 days in 6 wt. % hydrogel with GFOGER shown; see Fig. S4 for additional images). (c) At time points of interest, confocal z-stacks were analyzed in three dimensions to determine the size (volume) and morphology (shape factor) of single cells and clusters as quantitative measures of the cell response. Elongated or irregular shaped, spread cells have a lower shape factor, whereas spherical clusters have a higher shape factor.

We first examined the effect of the matrix density and modulus on the formation and growth of breast cancer spheroids from single cell suspensions within different synthetic matrix compositions. T47D and MDA-MB231 cells were encapsulated in 6 and 10 wt. % hydrogels containing 2 mM GFOGER, RGDS, or IKVAV and cultured for 10 days. To quantitatively assess the cell response, cells were immunostained (f-actin, cytoskeleton; DNA, nuclei), and the cell or cluster size and morphology were analyzed in three dimensions using confocal microscopy [Fig. 2(a)]. Cells initially began as a single cell suspension, which was consistently achieved with light-triggered formation of the hydrogel, and spread or formed multicellular spheroids over time. Further, cells remained confined to the top layer in which they were originally encapsulated throughout culture [Figs. 2(b) and S4). Analysis of the size (volume, *μ*m^3^) and morphology (shape factor) of these clusters and cells allowed identification of differences in the cell response within these discrete microenvironments of different moduli [Fig. 2(c)]. Specifically, cell elongation is indicative of a mesenchymal, migratory cell phenotype and was quantified with the shape factor (smaller shape factor = more spread), and the increased spheroid size is indicative of cell proliferation and growth and was quantified with the object volume (larger volume = more proliferation).^26,54–56^ Roughly, an object volume on the order of ∼4000 *μ*m^3^ was representative of a single cell, and an object volume on the order of ∼40 000 *μ*m^3^ was representative of a cluster of approximately 10–20 cells, based on the observed size of a single size and the average number of cells observed per cluster. An object shape factor of ∼0.5 was representative of elongated cells and clusters with highly irregular shapes (e.g., protruding cells) and ∼0.8 was representative of spherical cells and clusters. As discussed in depth below, cells generally exhibited a more active behavior (large clusters = T47D, irregular shape = MDA-MB-231) in the lower matrix density environments (6 wt. %), particularly with RGDS or GFOGER, when compared to the higher matrix density environments (10 wt. %), particularly with IKVAV (small clusters = T47D, spherical morphology = MDA-MB-231).

**FIG. 3.**
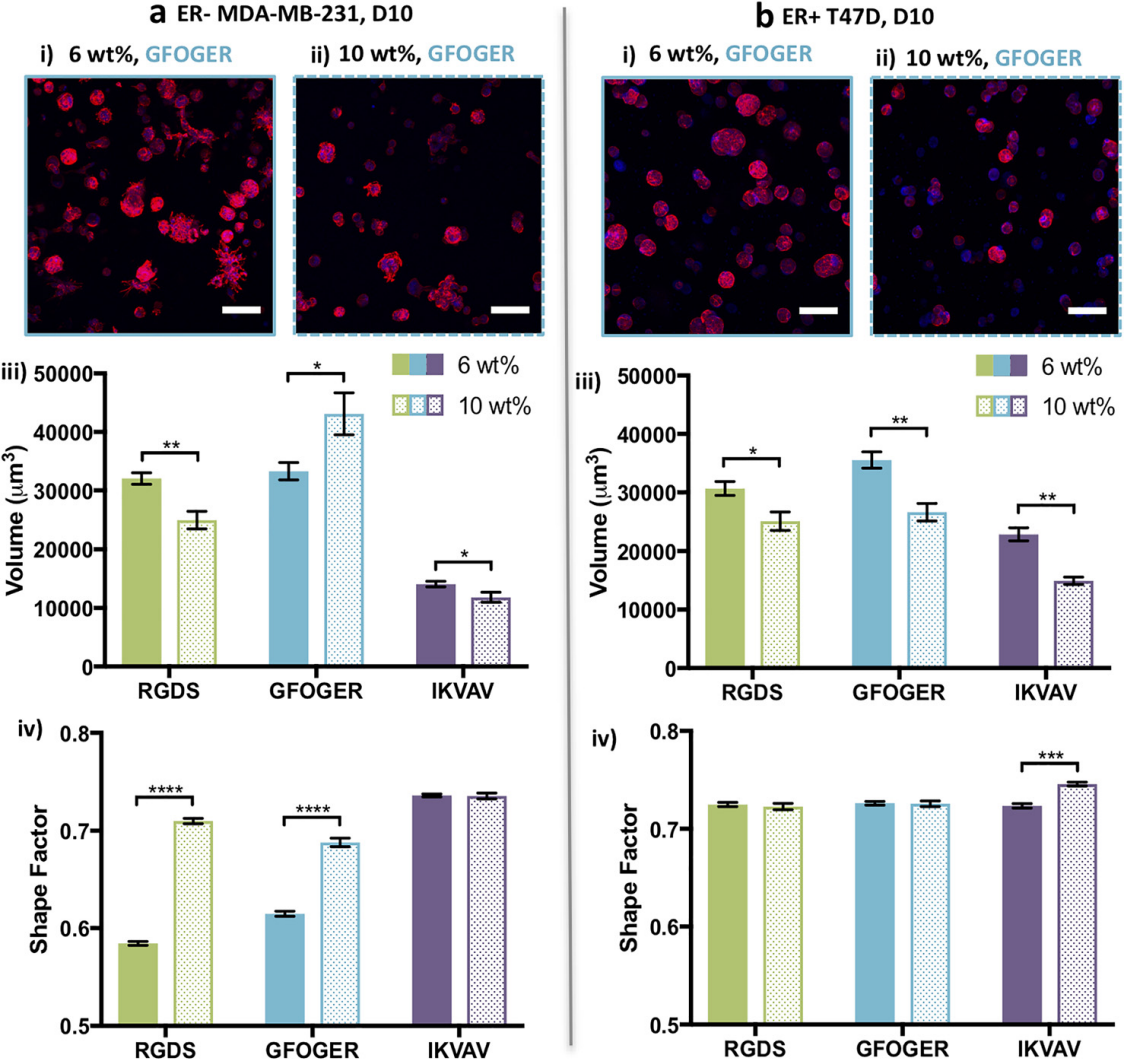
Effects of matrix density on cell responses within synthetic matrices. (a) For ER− MDA-MB-231 cells on day 10, significant differences in the cluster size were observed between (i) 6 and (ii) 10 wt. % matrices, with both single cells and cell clusters found within the same sample. (iii) Decreased cluster volume was observed with increasing matrix density for RGDS and IKVAV, whereas, importantly, the opposite was observed for GFOGER. (iv) Elongated cells (lower shape factor) were observed with lower matrix density and more spherical cells within higher matrix density with RGDS and GFOGER. Cells were rounded within both low and high density matrices containing IKVAV. (b) For T47D cells at day 10, significant differences in the cluster size were observed between (i) 6 and (ii) 10 wt. % environments, with (iii) decreased cluster volume for all peptides and (iv) increased spherical shape with IKVAV with increasing matrix density. Representative images shown for (i) 6 wt. % and (ii) 10 wt. % with 2 mM GFOGER (Z-stack projection, scale bar = 100 *μ*m, F-actin = red, and Nuclei = blue). The data shown illustrate the mean (n = 3 gels for N = 2 experiments with 6 wt. % conditions and n = 3 gels for the N = 1 experiment with 10 wt. % conditions; ≥50 objects/hydrogel counted) with error bars showing the standard error and statistical differences determined using Student's t-test (*p < 0.05, **p < 0.01, ***p < 0.001, and ****p < 0.0001).

For both “soft” and “stiff” matrices containing various peptides (6 and 10 wt. % environments, respectively), the ER− MDA-MB-231 cell line remained primarily as single cells or as small clusters, analyzed after 10 days in culture [Fig. 3(a)]. Importantly, larger clusters were observed in 10 wt. % environments containing GFOGER, whereas smaller cell clusters were observed in 10 wt. % environments containing RGDS or IKVAV in comparison to 6 wt. %. This observation suggested increased proliferation despite the need to degrade more of the synthetic matrix in the 10 wt. % GFOGER environment, which was further examined as detailed below. Additionally, the shape of cells and clusters in the 6 wt. % and 10 wt. % environments was highly differential. A more spherical morphology was observed in the 10 wt. % environments containing GFOGER or RGDS compared to the 6 wt. % environments, indicating that the more dense matrix restricted spreading that is characteristic of mesenchymal activation (supplementary material Table 1), whereas less spreading was observed in both 6 and 10 wt. % with IKVAV. These quantitative analyses of the volume and shape of cell clusters demonstrated the importance of both the matrix density and the integrin binding sequence in the cell response to the matrix.

T47D, a luminal A ER+ cell line, is generally considered less aggressive than ER− MDA-MB-231 cells and forms spherical clusters in both the 6 and 10 wt. % environments as measured by the shape factor [Fig. 3(b)]. However, higher matrix density was observed to consistently result in lower volumes when comparing clusters in the 10 wt. % environments with those in the 6 wt. % hydrogels (supplementary material Table 2). These data suggest that high matrix density reduced growth of the T47D spheroids within 10 wt. % environments.

To better understand the origins of the differential cluster volume for both ER− MDA-MB-231 and ER+ T47D cells, we performed cell cycle analysis for cells cultured in different matrix densities with the collagen mimic GFOGER. The effects of matrix density on cell proliferation and cell cycle at early and late times in 3D culture were assessed using flow cytometry. While MDA-MB-231 cells were observed to be proliferating within both wt. % matrices, notably, an increased cell population in the G2/M phase and the S phase was observed with increasing matrix density on day 3 and day 10 (Fig. S7). In contrast, for ER+ T47D cells, the cell population of the G0/G1 phase increased for both wt. % matrices on day 3 and day 10 (Fig. S7), indicating decreased cell proliferation or arrested growth phase over time (Fig. S7). These differences in cell proliferation support the observed differences in the cluster volume for the different breast cancer cell subtypes in response to matrix density.

### Integrin-binding peptides drive cell responses in synthetic matrices

Proteins and factors to which cells bind within the native microenvironment are thought to be key in the progression of breast cancer.^2,3,17^ In particular, remodeling of the ECM by stromal cells leads to an increase in the collagen content, which correlates with tumor growth and metastasis.^4,5^ To investigate how mixtures of matrix cues influence cell responses in these more complex environments, cells were cultured in matrices containing combinations of RGDS, GFOGER, and IKVAV. For these studies, we focused on the 6 wt. % matrices that were more permissive to cell growth and had moduli similar to healthy mammary tissue. Here, we aimed to test our overarching hypothesis that increased collagen and fibronectin content within a laminin-rich matrix, as observed during tumor growth and disease progression, differentially influenced cell response. Specifically, cells were cultured in 6 wt. % matrices rich in IKVAV (1.9 or 1.5 mM) and containing different concentrations of RGDS and GFOGER (0.05 mM or 0.25 mM each) toward mimicking aspects of the increased collagen and fibronectin content in the native environment during progression.^4,5^ The cell response to these mixtures of peptides was then compared with that observed with individual peptides and no peptide, where cells encapsulated in 6 wt. % matrix compositions were immunostained [F-actin, 4′,6-diamidino-2-phenylindole (DAPI)] after 10 days in culture, and the shape factor and volume were analyzed.

**FIG. 4.**
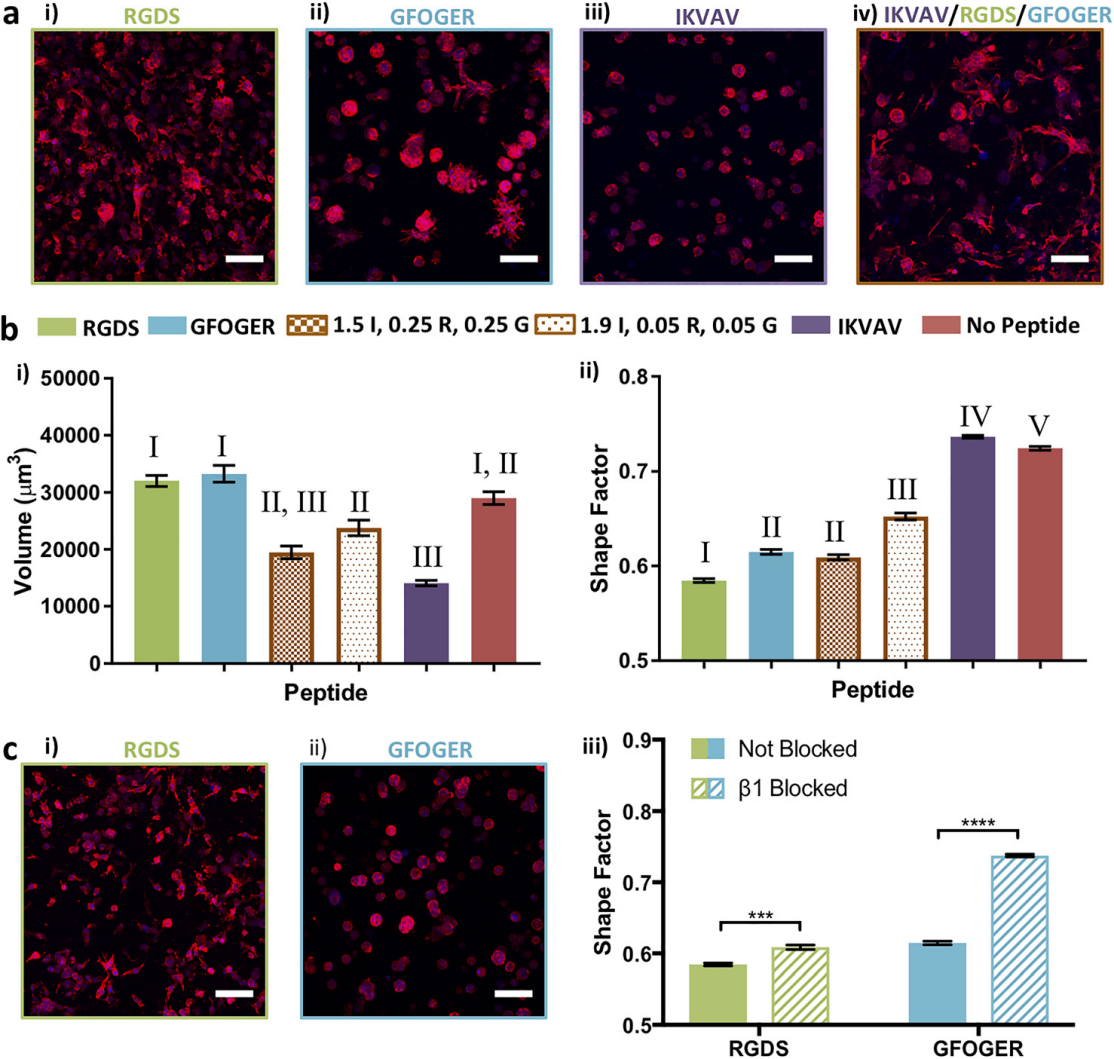
Response of MDA-MB-231 cells to individual and mixtures of biochemical cues. (a) Different responses were observed for encapsulated ER− MDA-MB-231 to different biochemical cues: (i) 2 mM RGDS (R), (ii) 2 mM GFOGER (G), (iii) 2 mM IKVAV (I), and (iv) 1.5 mM IKVAV/0.25 mM RGDS/0.25 mM GFOGER in low matrix density (6 wt. %) at day 10. (b) Quantitatively, for ER− MDA-MB-231 cells, (i) statistically larger average cell/cluster sizes were observed in response to RGDS or GFOGER relative to IKVAV, and titration of small amounts of GFOGER and RGDS into a matrix rich in IKVAV commensurately increased the volume. Further, a statistically decreased volume was observed in response to IKVAV relative to no peptide. (ii) Statistically significant differences in cell spreading (shape factor) were observed in response to RGDS, GFOGER, 1.9/0.05/0.05 mM IKVAV/RGDS/GFOGER, IKVAV, and no peptide. Further, a significant difference in the shape factor was observed between the 1.5/0.25/0.25 and 1.9/0.05/0.05 mM IKVAV/RGDS/GFOGER compositions. (c) Blocking of β1 integrin led to significantly less spreading for ER− MDA-MB-231 in synthetic matrices (6 wt. %) containing (i) RGDS or (ii) GFOGER and (iii) with the greatest response observed in the presence of GFOGER. The data shown are for day 10 in culture and illustrate the mean (n ≥ 3; ≥50 objects/hydrogel counted) with error bars showing the standard error and statistical differences determined (b) by one-way analysis of variance (ANOVA) with Tukey's Multiple Comparisons (different Roman numerals are statistically different with p < 0.01; Roman numerals are statistically the same; the full list of p-values is given in supplementary material Table S1) and (c) using Student's t-test (*p < 0.05, **p < 0.01, ***p < 0.001, and ****p < 0.0001). Representative images (Z-stack projection, scale bar = 100 *μ*m, F-actin = red, and Nuclei = blue).

Notable differences in the cluster volume and shape were qualitatively observed for MDA-MB-231 cells when even small amounts of GFOGER and RGDS were mixed with IKVAV [Fig. 4(a)]. Quantitatively, statistically significant differences in the volume were observed between 0.05 mM of GFOGER and RGDS in an IKVAV-rich matrix and IKVAV, RGDS, or GFOGER alone [Fig. 4(b), supplementary material Table 1]. Interestingly, a statistically decreased cluster volume was observed between IKVAV alone and no peptide, supporting the suppressive effect of this integrin sequence on cluster growth and its incorporation into the synthetic matrix. Further, MDA-MB-231s exhibited significantly less spreading (larger shape factor) in matrices with IKVAV alone in comparison to no peptide and all other peptide compositions. Importantly, a dose dependent response of increased spreading was observed with increasing concentrations of GFOGER and RGDS within an IKVAV-rich matrix with the most spreading (lowest shape factors) observed for GFOGER and RGDS alone.

Both the RGDS and GFOGER peptides are known to bind integrin β1 (ITGB1), which is associated with proliferation and metastasis of breast cancer cells and a poor prognosis in patients.^49–51,57^ Toward identifying whether integrin β1 played a role in the response observed for cells cultured in matrices with GFOGER and RGDS, binding to integrin subunit β1 was blocked. Specifically, prior to encapsulation in 6 wt. % matrices with 2 mM GFOGER or RGDS, cells were incubated with an anti-integrin-β1 antibody (100 *μ*g/ml), previously described for blocking normal and tumorigenic breast epithelial cells cultured in naturally derived matrices (Matrigel, Collagen I).^58^ To maintain blocking throughout the 10-day culture, culture medium was supplemented with anti-integrin-β1.

As shown in Fig. 4(c), β1-blocked MDA-MB-231 cells exhibited significant differences in response to both GFOGER and RGDS peptides. A spherical cluster morphology was observed in both GFOGER- and RGDS-containing hydrogels, which were statistically different from the response of non-blocked cells in their respective control environments [Fig. 4(c) and supplementary material Table 3]. These data support the key role of β1 integrin binding in cell response to both GFOGER and RGDS. Note that the spherical morphology of blocked cells within hydrogels containing GFOGER was statistically similar to the morphology in hydrogels containing IKVAV, whereas the morphology of blocked cells in the RGDS-containing hydrogels remained more irregular (Fig. S8), suggesting that other integrins targeted by the RGDS peptide (e.g., αvβ3) may also play a role in the observed cell response to these synthetic matrices.

**FIG. 5.**
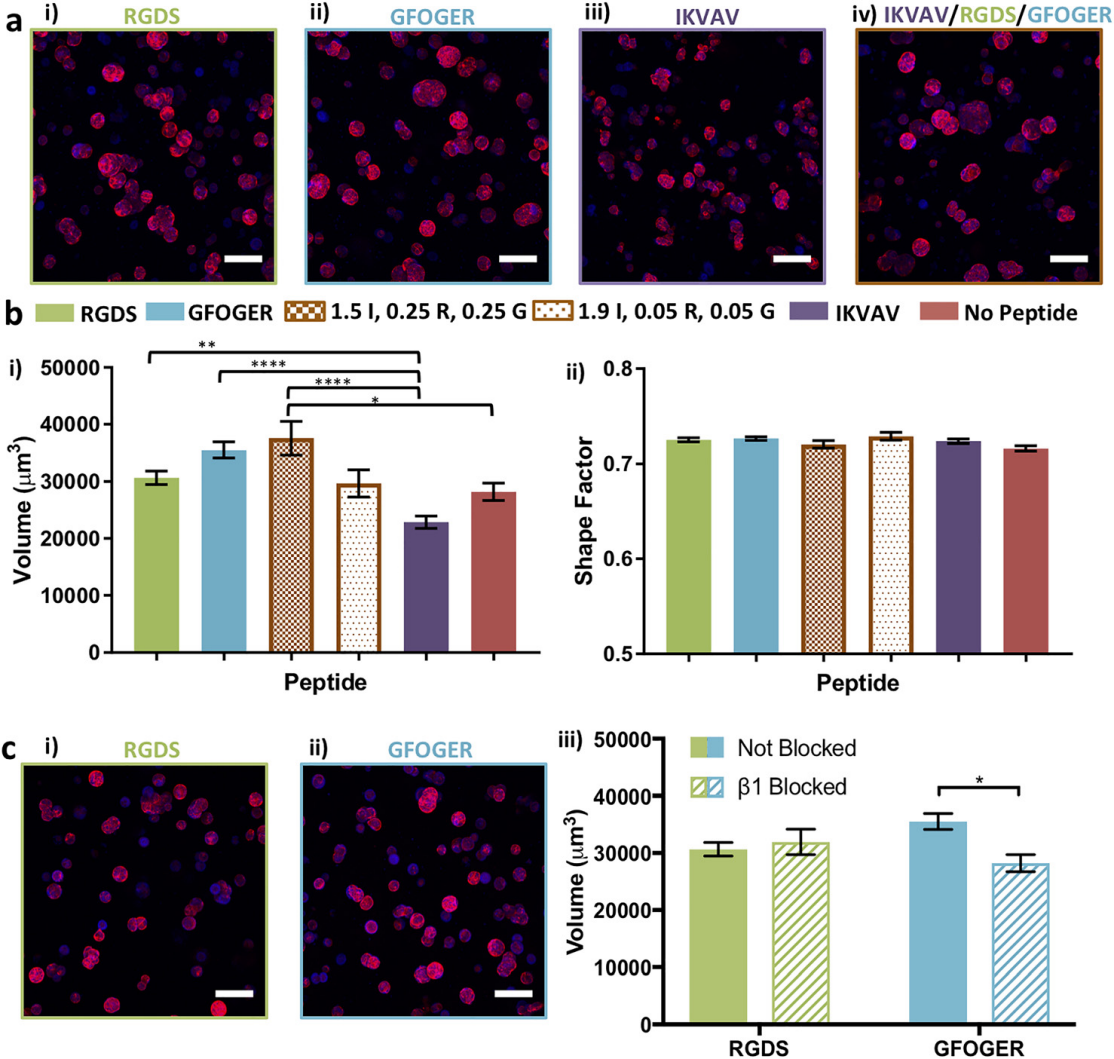
Response of T47D cells to individual and mixtures of biochemical cues. (a) Different responses were observed for encapsulated ER+ T47D in response to different biochemical cues: (i) 2 mM RGDS (R), (ii) 2 mM GFOGER (G), (iii) 2 mM IKVAV (I), and (iv) 1.5 mM IKVAV/0.25 mM RGDS/0.25 mM GFOGER in low matrix density at day 10. (b) Quantitatively, for ER+ T47D cells, (i) statistically larger average cluster volumes were observed in response to RGDS, GFOGER, or a mixture of peptides 1.5/0.25/0.25 mM IKVAV/RGDS/GFOGER relative to IKVAV, where the smallest clusters were observed. (ii) No significant difference in the shape factor was observed for ER+ cells, consistent with the qualitative observations of spheroids in all compositions. (c) Blocking of β1 integrin for ER+ T47D cells in synthetic matrices (6 wt. %) containing (i) RGDS or (ii) GFOGER, which led to (iii) significantly decreased growth in response GFOGER but did not impact growth in response to RGDS (day 10 in culture). The data shown illustrate the mean (n ≥ 3; ≥50 objects/hydrogel counted) with error bars showing the standard error and statistical differences determined (b) by one-way ANOVA with Tukey's Multiple Comparisons and (c) using Student's t-test (*p < 0.05, **p < 0.01, ***p < 0.001, ****p < 0.0001). Representative images (Z-stack projection, scale bar = 100 *μ*m, F-actin = red, and Nuclei = blue).

As shown in Fig. 5, the response of T47D cells encapsulated with mixtures of peptides was similarly investigated. While spheroids were observed in all conditions [Fig. 5(a)], significantly larger cluster volumes were observed in hydrogels containing RGDS, GFOGER, and 0.25 mM GFOGER and RGDS in an IKVAV-rich matrix when compared to a matrix with IKVAV [Fig. 5(b), supplementary material Table 2], indicating a dose dependent response in the growth of T47D cells to mixtures of biochemical cues. To examine the role of β1 in the response of cells to matrices with GFOGER or RGDS, β1-blocking was performed on T47D cells prior to encapsulation and throughout culture. Significantly smaller clusters were observed for β1-blocked cells in matrices with GFOGER but not with RGDS, relative to the respective non-blocked controls [Fig. 5(c), supplementary material Table 3], supporting the importance of β1 binding in the T47D cell response to GFOGER. Note that the magnitude of differences in cell responses observed for T47D cells in comparison to MDA-MB-231 cells suggests more broadly that ER+ T47D cells were less responsive to differences in the matrix composition than ER− MDA-MB-231 cells within the ranges probed here and may depend more on cell-cell interactions, where robust expression of E-cadherin was observed for these luminal A cells (Fig. S5).^26^

### Breast cancer cells exhibit differential responses in proliferation and gene expression to matrix composition over time

A better understanding of how breast cancer cells initially respond to matrix compositions, as well as over time, can provide insights into key regulators of cell responses to specific microenvironment cues. Of particular interest is how cancer cells respond to laminin-rich vs. collagen-rich microenvironments: for example, during cancer development and progression, the epithelium is remodeled, undergoing degradation followed by deposition and crosslinking of collagens and transitioning from a soft, laminin-rich matrix to a stiff, collagen-rich matrix that influences the maladaptive activities of cancer cell.^4^ Further, in our studies, large quantitative differences in cell responses were observed for relevant mimics of laminin and collagen (IKVAV vs. GFOGER, respectively) at day 10 in culture. To investigate these differences further, we studied cell responses over time to 6 wt. % matrices containing IKVAV or GFOGER using imaging, metabolic, and bioinformatics analyses.

As noted earlier in viability analyses (Fig. 1), cells in these 3D cultures began as single cells evenly dispersed throughout and surrounded by the synthetic matrix. Indications of cell proliferation were observed even at early times in 3D culture, where expression of Ki-67 (a nuclear marker associated with the cell cycle and proliferation) was observed for both cell types (Fig. S9). This cell proliferation was confirmed and quantified using not only measurements of the increased cluster size (Figs. 3 and S9) but also the commensurate increase in metabolic activity from day 3 to day 10, indicating both cell viability and increased numbers of cells (Fig. S9). To verify that these observations of viability and proliferation were not strictly limited to MDA-MB-231 and T47D cells, two other ER+ cell types (luminal A ZR-75–1 and luminal B HER2+ BT474) were encapsulated and cultured in 6 wt. % matrices with GFOGER: increased metabolic activity was similarly observed over time (Fig. S6), indicating cell viability and proliferation and, more broadly, supporting the relevance of the observations and utility of the 3D culture system.

**FIG. 6.**
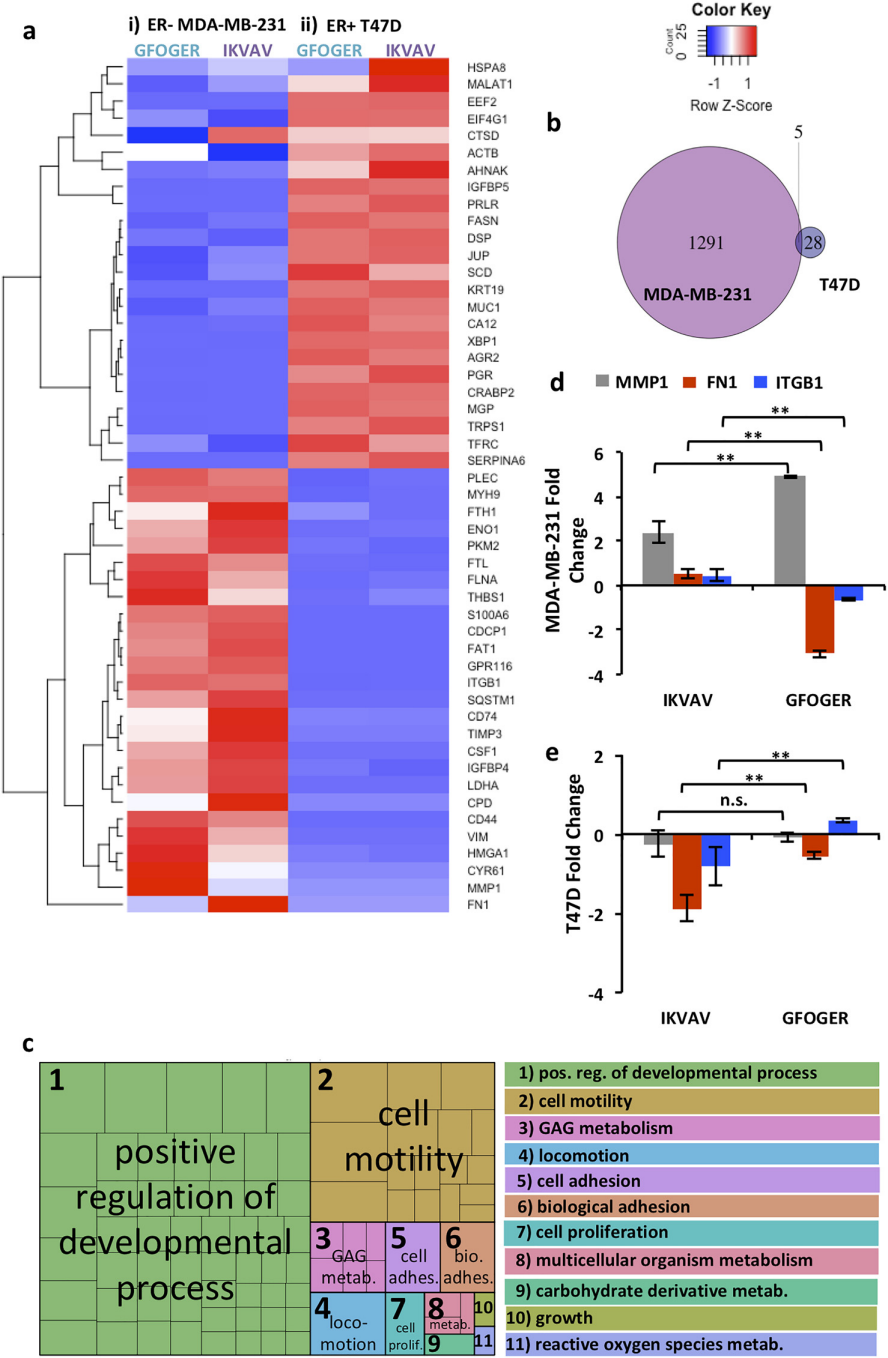
Probing response of cells to biochemical cues with bioinformatics tools. Transcriptome level responses of (i) encapsulated ER− MDA-MB-231 and (ii) ER+ T47D to a soft matrix (6 wt. %) rich in collagen mimic (2 mM GFOGER) or laminin mimic (2 mM IKVAV) were examined using next generation sequencing. (a) Heatmap of average counts per million (cpm) for each condition on day 3, where top 50 genes with most variance are shown and rows are scaled to a mean of 0 and a standard deviation of 1 (z-score) and reordered via clustering (shown on the left side). (b) Venn diagram showing the number of genes differentially expressed at a minimum fold change of 2 and FDR < 0.05 on day 3 for MDA-MB-231 GFOGER vs. IKVAV (purple) and T47D GFOGER vs. IKVAV (blue). (c) REVIGO tree map of gene ontology terms for enriched biological processes based on the MDA-MB-231 differentially expressed genes. The boxes within the rectangle on the left are colored according to the functional category, and the size of each rectangle is proportional to the corrected p-value for that category, where a legend is provided on the right. (d) Selected genes related to enriched GO terms for biological processes relevant for cell-matrix interactions, proliferation, and motility were examined using qRT-PCR: MMP1 (grey), FN1 (red), and ITGB1 (blue) for the 6 wt. % matrix containing GFOGER or IKVAV. For MDA-MB-231 cells, significantly increased expression of MMP1 and decreased expression of FN1 and ITGB1 were observed in GFOGER relative to IKVAV. (e) For T47D cells, significantly increased expression of ITGB1 was observed in GFOGER relative to IKVAV, whereas significantly decreased expression of FN1 was observed in IKVAV relative to GFOGER. [Data represent the mean for n = 3, fold change calculated as −ΔΔCt of each gene for the 3D culture condition relative to the 2D culture growth condition, and non-parametric two-sided Mann Whitney determined significance (*p < 0.05 and **p < 0.01)].

Responses of MDA-MB-231 and T47D cells to these matrix compositions at early times in 3D culture were investigated further using next generation sequencing (RNA-seq). The top 50 genes with the most variance in expression across these samples, 6 wt. % matrices with IKVAV or GFOGER, were examined [Fig. 6(a)]. Broadly, the heat map of these genes revealed more variance in the response of basal MDA-MB-231 cells to the different matrix compositions than luminal A T47D cells at this early time in cell culture. Additionally, as expected based on their respective subtypes, significant differences in gene expression were observed between these two cell lines independent of the matrix composition. Analysis of differential expression in GFOGER vs. IKVAV matrices at day 3 in culture confirmed these observations: 1296 significantly differentially expressed genes for the ER− MDA-MB-231 cells and only 33 for the ER+ T47D cells with minimal overlap [Fig. 6(b), Tables S2 and S3], where genes with a significant change were identified as those with a fold change of at least 2 and a false discovery rate (FDR) of less than 0.05. Interestingly, for MDA-MB-231 cells, significant differences in expression were observed for genes associated with matrix remodeling and cell-matrix interactions that are important in progression and metastasis, including decreased tissue inhibitor of metalloproteinase-3 (TIMP3) and fibronectin-1 (FN1) and increased matrix metalloproteinase-1 (MMP1) and a cell surface adhesion receptor (CD44), in response to GFOGER relative to IKVAV.^59–63^

To better understand any underlying differences in biological processes associated with these genes, we performed gene ontology (GO) enrichment analysis for the 1296 genes that met our differential expression criteria for the ER− MDA-MB-231 cells in the GFOGER vs. IKVAV matrices. Note that the number of differentially expressed genes for the ER+ T47D cells was too small to perform this analysis. We identified 192 GO Biological Process terms with a Benjamini-Hochberg corrected p-value < 0.05 (Table S7), which are summarized in a REVIGO tree map [Fig. 6(c), Table S8]. These analyses revealed enrichment of biological processes associated with developmental processes and cell adhesion, motility, and proliferation, more broadly confirming observations made based on differential expression of individual genes. These transcriptome level analyses, in concert with our phenotypic observations, supported that matrices with the collagen mimic promoted increased cell activation (e.g., matrix remodeling, proliferation, and motility) as compared to the laminin mimic for the ER− MDA-MB-231 cells.

To verify and validate observations from RNA-seq and enriched GO term analyses, we selected specific genes from the list of the top 50 genes with most variance that are associated with many of the enriched GO terms observed for MDA-MB-231 cells and examined changes in their level of expression. Specifically, using qRT-PCR, we examined expression of MMP-1 (MMP1) associated with enriched GO terms related to cell motility, locomotion, and developmental processes, and fibronectin (FN1) and integrin β1 (ITGB1) associated with enriched GO terms related to developmental processes, cell motility, cell adhesion, biological adhesion, cell proliferation, and growth. Expression of these genes was analyzed by comparing the fold change in response to different matrix compositions in 3D culture (6 wt. % GFOGER or IKVAV) with a growth control (2D culture on plates), and statistical differences in response between matrix compositions were then assessed.

For MDA-MB-231 cells, we observed significantly increased MMP-1 expression and decreased fibronectin and β1 integrin expression with the collagen mimic GFOGER compared to the laminin mimic IKVAV [Fig. 6(d)]. These observations of gene expression are consistent with and validate our RNA-seq and GO term analyses, as well as provide additional insights into β1 integrin expression. The increased expression of FN1 and β1 integrin in matrices with IKVAV compared to GFOGER suggests a role of matrix remodeling and cell-matrix interactions the survival and growth of MDA-MB-231 cells in the IKVAV-rich matrix, which were observed to be suppressive of cluster growth at the cellular level [Fig. 4(b)]. For T47D cells, significantly increased expression of ITGB1 was observed in GFOGER relative to IKVAV, whereas significantly decreased expression of FN1 was observed in IKVAV relative to GFOGER [Fig. 6(e)]. Taken together, these observations for MDA-MB-231 and T47D cells further support the importance of β1 integrin in cell responses to different matrix compositions. While β1 is known to play a role in breast cancer cell proliferation, whether this cell-matrix interaction promotes increased or decreased proliferation has been observed to be context dependent (e.g., dependent on cell-cell contact in 2D culture)^64^ and the approach established here provides future opportunities for more in-depth studies of this interplay in 3D culture.

## DISCUSSION

In this work, we established and utilized a well-defined, 3D synthetic culture system to investigate how combinations of biochemical and biophysical cues affect breast cancer cell responses toward understanding their role in disease progression. For these studies, cell lines were selected as prototypical examples of different breast cancer subtypes, specifically less aggressive luminal A and highly aggressive basal cells, which have different potential for growth, invasion, metastasis, and recurrence.^65–67^ Using this approach, we observed that more dense matrices (10 wt. % vs. 6 wt. %) restricted MDA-MB-231 spreading (as measured by the shape factor), while cluster growth (volume) in response to matrix density was dependent on the integrin binding ligand. Decreased cluster growth was observed in 10 wt. % matrices with RGDS and IKVAV relative to 6 wt. %, where similar observations have been made with other hydrogel-based synthetic ECMs.^29^ However, importantly, increased cluster growth was observed in 10 wt. % matrices with GFOGER relative to 6 wt. %, consistent with *in vivo* observations of collagen-rich ECMs of increased stiffness correlating with cancer progression.^68,69^ In contrast, T47D cluster growth (volume) was restricted in dense matrices regardless of the pendant peptide (RGDS, GFOGER, and IKVAV). Broadly, we hypothesize that restriction of cluster growth and spreading is due to increased matrix density (concentration of polymer and enzymatically degradable crosslinking peptide) proximate to encapsulated cells, which may require increased secretion of MMPs to degrade the surrounding network. The notable increase in cluster growth for MDA-MB-231 in 10 wt. % matrices with GFOGER relative to 6 wt. % was correlated with an increase in the growth phase of the cell cycle (percentage of cell population in G2/M and S phases). This increased proliferation in response to increased matrix density suggests a role of the matrix modulus (e.g., mechanotransduction) in addition to the effects of matrix density. With the culture system established here, alternative crosslinking peptides with faster or slower degradation in response to different MMPs could be investigated in future studies to further probe how local matrix degradation affects cell spreading and growth in matrices of different densities, where the photopolymerizable thiol-ene chemistry can also be exploited to explore the effects of heterogenous matrix properties (e.g., gradients).^70^

Different compositions of proteins are found within native tissues, depending on the tissue structure and function, and change over time as a result of remodeling that is correlated with disease progression. Several *in vivo* studies have demonstrated that cellular remodeling of native tissues creates a permissive environment for cancer progression due to increased deposition of fibronectin and collagen I and that the secretion of soluble factors, like TGFβ, stimulates collagen deposition within the tumor microenvironment.^4,71^ The study described here represents a primary investigation into how biochemical cues affect the breast cancer cell response within a fully synthetic, tunable 3D culture system. Specifically, we sought to test the hypothesis that a matrix enriched in collagen and fibronectin/vitronectin, mimicking aspects of the remodeled epithelium that is observed natively during tumor progression, would stimulate breast cancer cells relative to a matrix rich in laminin, mimicking aspects of a healthy mammary epithelium. Taking inspiration from these native environments, the effects of individual and combinations of integrin- and receptor-binding peptides found within these environments, RGDS, GFOGER, and IKVAV, were investigated.

In synthetic matrices containing RGDS or GFOGER, the highest levels of response were observed, with ER− MDA-MB-231 cells forming clusters with irregular morphology and ER+ T47D cells forming large spheroids relative to cells cultured with IKVAV. Different integrins targeted by each peptide and expressed by each cell line may play a major role in the different responses observed. Specifically, RGDS has been shown to target integrins αvβ3 and α5β1, amongst others, GFOGER primarily targets α1β1 and α2β1, and IKVAV primarily targets the laminin receptor. MDA-MB-231 is known to express αvβ3, α2β1, and α1β1;^72,73^ thus, the irregular morphology in GFOGER and RGDS is characteristic of mesenchymal activation and binding of related key integrins, where our blocking studies confirmed the importance of β1 binding. On the other hand, luminal A cells like T47Ds have been shown to express α2β1, but not αvβ3.^74,75^ The most significant difference in the T47D response was found between GFOGER and IKVAV, supporting that binding to β1 may play a role in the formation of larger cell clusters. Indeed, blocking of β1 integrin mitigated the growth response of T47Ds in matrices with GFOGER, confirming its importance. The response of T47D cells to RGDS was slightly less but not significantly different when compared to GFOGER; however, blocking of β1 integrin did not mitigate the growth response of T47Ds in matrices with RGDS, supporting the role of its promiscuous binding (e.g., αvβ3 and α5β1) in these observations.

Note that integrin binding has been implicated in regulating cellular processes associated with cancer progression. For example, αvβ3 has been implicated in the metastatic cascade by promoting invasion and adhesion within tissues,^76,77^ and β1 integrin has been shown to promote survival and proliferation of tumor cells in metastatic tissue sites.^78^ In particular, β1 has been a focus of numerous studies investigating cellular pathways that drive cancer cell proliferation and other activities. For example, proliferation of tumor cells mediated by β1 integrin signaling has been reported in naturally derived 3D culture environments, where β1 binding has been shown to induce phosphorylation of Src and focal adhesion kinase (FAK) and activate extracellular signal-related kinase (ERK).^79–81^ Our studies confirm the importance of β1 integrin in the growth of breast cancer cells in response to different matrix cues while providing new insights into the differential response of several breast cancer subtypes to specific matrix compositions at both the cellular and gene levels.

Building upon these findings, future investigations incorporating individual and mixtures of peptides could be performed with additional sequences derived from the same ECM proteins but targeting different integrins and receptors (e.g., YIGSR instead of IKVAV, Laminin mimic)^52,82^ or peptides that have been designed with increased specificity for a single integrin (e.g., PHSRN-RGDS and α5β1).^83^ Additionally, this system may be photopatterned, as previously described, where peptides are diffused into the matrix, attached via a second dose of light under a photomask, and rinsed out post-polymerization.^47,48^ In the future, one could envision patterning individual and combinations of peptides to study the response of breast cancer cells to heterogeneous matrix cues (e.g., directional proliferation, spreading, or migration in response to a gradient^84^ or from one patterned region to the next). Other soluble factors secreted by niche cells (e.g., hMSCs and fibroblasts) may also be investigated using the approaches established here to identify critical combinations of cell-cell and cell-matrix which more broadly regulate cell responses, particularly for T47D and other ER+ cancer cells.

Finally, the effects of biochemical cues, specifically environments rich in collagen vs. laminin mimic, were examined further both initially and over time. Starting from single cells, increased proliferation was observed for both MDA-MB-231 and T47D cells in response to GFOGER relative to IKVAV. Importantly, other cell lines from the same or different breast cancer subtypes, ER+ luminal A ZR-75–1 and ER+ HER2+ luminal B BT474, exhibited similar responses of viability and growth to matrices with GFOGER, supporting the relevance of observations within the 3D culture system. Broad analysis of gene expression for MDA-MB-231 and T47D cells using RNA-seq, validated by qRT-PCR, further revealed differential responses of these cell lines to the matrix composition. Enriched genes and biological processes associated with cell-matrix interactions, proliferation, and motility were observed for MDA-MB-231 cells, and differential expression of β1 integrin was observed between MDA-MB-231 and T47D cells. These well-defined synthetic matrices combined with quantitative imaging and bioinformatics analysis workflows now provide opportunities for future investigations of changes in the cell response between early and late times in culture for comparison to clinical datasets and potential mechanistic studies and evaluation of therapeutics.

## CONCLUSION

The culture of breast cancer cells (MDA-MB-231, T47D) within a tunable and fully synthetic hydrogel-based matrix was described for the investigation of cell response to key mechanical and biochemical cues. Precise control over both chemical and mechanical properties allowed investigation of both individual and combinations of cues within discrete microenvironments. Dense matrices were generally shown to restrict the growth and spreading of both MDA-MB-231 and T47D cell lines, with the notable exception of increased growth of MDA-MB-231 cells in dense matrices rich in collagen mimic GFOGER, which correlates with *in vivo* behavior. The response to individual and mixtures of biochemical cues (receptor-binding peptides) incorporated within networks was also studied, with collagen-mimetic (GFOGER) and fibronectin/vitronectin-mimetic (RGDS) peptides resulting in increased levels of response for both cell lines compared to the laminin-mimetic (IKVAV) peptide. Blocking binding to integrin β1 was shown to reduce growth of T47D clusters in response to GFOGER and spreading of MDA-MB-231 in response to RGDS or GFOGER, indicating that binding to integrin β1 was important to responses observed with these synthetic matrices and consistent with the reported importance of β1 integrin and, more broadly, collagen in cancer progression. Finally, examination of cells in culture over time using both imaging and bioinformatics techniques demonstrated how the matrix composition influenced cell proliferation and matrix remodeling related processes and supported the promise for these approaches for future investigations of the cell response to changes in microenvironmental cues (e.g., incorporation of cleavable pendant peptides, addition of growth factors, and gradients in matrix cues). These studies represent a unique and useful approach to examining the breast cancer response within a synthetic, 3D matrix. In future work, this system could be used in a wide range of applications, including primary cell cultures and co-culture with niche cells, mechanistic studies for identification of new therapeutic targets, and evaluation of new therapeutics for improved treatment strategies to prevent cancer progression.

## METHODS

### Macromer and initiator synthesis

Macromers for hydrogel formation were synthesized following the established methods.^47,48^ A 4-arm poly(ethylene glycol) thiol (PEG4SH, 20 kDa MW), the “backbone” within the hydrogel structure, was synthesized via a 3-step reaction as described previously (Fig. S10).^47,48^ Briefly, peptides containing alloxycarbonyl (alloc)-protected lysines, providing a reactive vinyl, were synthesized via solid phase peptide synthesis (MBHA rink amide resin, Novabiochem; high-swelling Chemmatrix resin, Protein Technologies, for the GFOGER sequence) on a Protein Technologies PS3 synthesizer using standard Fmoc chemistry (0.25 mmol scale), where all amino acids were double coupled. Peptides were cleaved from resin [4 h in 95% v/v trifluoroacetic acid (Acros Organics), 2.5% v/v triisopropylsilane (Acros Organics), and 2.5% v/v water with 5% w/v dithiothreitol (DTT) (Research Products International Corporation) to prevent disulfide formation and 2.5% w/v phenol (Sigma Aldrich) to protect tryptophan (W)], precipitated in ice cold ethyl ether, purified by high performance liquid chromatography (XBridge C18 column with a linear water-acetonitrile (ACN) gradient; water: ACN 95: 5 to 45: 5; 1.17% change in water per minute), and lyophilized. Successful synthesis of the enzymatically degradable crosslink Ac-K**K(alloc)**G[GPQG↓IWGQ]G**K(alloc)**K (Pep2Alloc) and pendant peptides **K(alloc)**(PEG_2_)_2_W(PEG_2_)IKVAV (laminin mimic, IKVAV), **K(alloc)**GWGRGDS (fibronectin/vitronectin mimic, RGDS), and **K(alloc)**G(POG)_4_FOGERG(POG)_4_G (collagen mimic, GFOGER) was confirmed via mass spectrometry (Figs. S11–S14). The photoinitiator, lithium acylphosphinate (LAP), was also synthesized as previously described.^47,48^

### Hydrogel synthesis and characterization

Hydrogels were polymerized, and their mechanical and biochemical properties were characterized using established methods.^47,48^ Briefly, PEG4SH, Pep2Alloc, and pendant peptides (IKVAV, RGDS, or GFOGER) were dissolved in phosphate buffered saline (PBS, Invitrogen) supplemented with 1% penicillin streptomycin (PS, Invitrogen) and 0.5 *μ*g/ml fungizone (FZ, Invitrogen). Hydrogel precursor solution, containing 6 or 10 percent PEG by weight (wt. %), was prepared by mixing PEG4SH with 2 mM pendant peptides and stoichiometric ratios of Pep2Alloc (final [SH] = [Alloc]). The precursor was pipetted into the tip of a sterile, cut syringe (1 ml). Collimated light at 365 nm and 10 mW/cm^2^ (Inpro Technologies collimating adaptor, Exfo Omnicure Series 2000) was applied. After polymerization, hydrogels were removed from syringe tips and placed in appropriate buffer (PBS, growth medium or Ellman's reaction buffer) for subsequent analysis.

To confirm the time needed for hydrogel formation, hydrogel formation was characterized by rheometry (TA AR-G2 with light attachment), performing a time sweep during photopolymerization of 6 wt. % PEG and 10 wt. % hydrogel precursor solutions (10 mW/cm^2^ at 365 nm); the time required for complete gelation was estimated to be the time at which the rate of change of G′ was within 10% of the maximum rate of change. To confirm that functional group conversion was not impacted by the different pendant peptides (RGDS, IKVAV, or GFOGER) incorporated within the hydrogel, Ellman's assay was performed to check free thiol concentration post-polymerization, as previously described.^47,48^ To establish the mechanical properties of the resulting hydrogels, modulus measurements were performed on 6 and 10 wt. % hydrogels polymerized for 1 min with either 2 mM excess free thiol or 2 mM of pendant peptide and equilibrium swollen in culture medium at 37 °C overnight. Swollen hydrogels were placed between parallel plates (20 mm geometry and Peltier Plate) on a rheometer (TA AR-G2) and compressed to a normal force of 0.01 N to prevent slip. Strain- and frequency-sweeps were performed to determine the linear viscoelastic (LVE) regime, and 2% strain and 2 rad/s frequency within the LVE regime were selected to perform measurements of modulus for equilibrium swollen hydrogels (Fig. S1). Young's, or elastic, modulus E was calculated from the measured swollen shear modulus using rubber elasticity theory, where E ≈ 3G for Poisson's ratio of ν = 0.5.^47^

### Cell culture and collection

MDA-MB-231 and T47D breast cancer cells (passages 16–24, ATCC), as well as ZR-75–1 and BT474 breast cancer cells (passages 4–8, ATCC), were cultured on tissue culture poly(styrene) in Dulbecco’s Modified Eagle’s Medium (DMEM) (Corning Cellgro) supplemented with 10% v/v fetal bovine serum (FBS, Invitrogen) and 1% PS. Growth medium was replaced every 48–72 h during culture. At 80% confluence, cells were passaged (1:4) or collected for experiments. Specifically, to collect cells for experiments, cells were removed from plates (trypsin/EDTA, 5 min, Corning Cellgro), counted using a hemocytometer, and aliquoted for the desired number of cells based on the experiment and hydrogel compositions to be tested. The aliquots were centrifuged (1200 rpm, 3 min), and the cell pellet was retained for subsequent treatment with blocking antibodies or mixing with the hydrogel precursor for encapsulation.

### Cell encapsulation

Cell encapsulation was performed according to a modified version of a published protocol.^47,48^ Briefly, MDA-MB-231, T47D, ZR-75‐1, and BT474 cells were collected, centrifuged, and re-suspended in the hydrogel precursor (6 or 10 wt. %, 2 mM total pendant peptide) at 5000 cells/*μ*l. For viability experiments, cell suspensions were pipetted into the tips of sterile cut syringes (20 *μ*l) and irradiated with light (365 nm, 10 mW/cm^2^) for 1 min. Post-polymerization, hydrogels were placed in a sterile, untreated 48-well plate and rinsed with fresh culture medium. Culture medium was replaced every 48–72 h. To prevent potential nutrient or oxygen gradients from encapsulation in thick (20 *μ*l) hydrogels during longer culture periods, cells were encapsulated using the following method: (i) 15 *μ*l of cell-free hydrogel precursor was polymerized in a sterile, cut syringe tip mold (1 min, 365 nm, 10 mW/cm^2^) and (ii) 5 *μ*l of cells suspended in the hydrogel precursor (5000 cells/*μ*l) was pipetted on top of the 15 *μ*l base and polymerized with a second dose of light (1 min, 365 nm, 10 mW/cm^2^). Post-polymerization, hydrogels were placed in sterile, untreated 48-well plates with the cell layer on top and rinsed 2 times with fresh culture medium. Culture medium was replaced every 48–72 h during the course of experiments. This “on-top” encapsulation method was used for immunostaining experiments investigating the cell response to matrix density, individual and peptide combinations, and β1 blocking, including all 10-day time course experiments. Note that since well-established human cell lines available in the public domain were used in these studies, ethics approval is not required.

### Viability assays

Viability of MDA-MB-231, T47D, ZR-75‐1, and BT474 cells encapsulated in hydrogels was determined using a live/dead viability/cytotoxicity kit (Invitrogen). Cells were encapsulated in hydrogels and cultured for 1 and 3 days, 6 and 10 wt. % hydrogels containing 2 mM RGDS for MDA-MB-231 and T47D cells as shown in Fig. 1 (n = 3 hydrogels). At days 1 and 3, hydrogels were rinsed (medium 3 × 10 min, PBS), stained (1 × 45 min, 4 *μ*M ethidium homodimer, 2 *μ*M calcein AM in PBS), and rinsed (3 × 10 min, PBS). Samples were immediately imaged on a Zeiss LSM 780 confocal microscope. Three z-stacks (100 images per stack, 2 *μ*m spacing) were taken per hydrogel (n = 3), for a total of 9 images. To quantify percent viability, live (green) and dead (red) cells were counted in each image z-projection.

### Immunostaining experiments

3D immunostaining experiments were conducted to investigate the cell response within synthetic microenvironments. Blocking and permeabilization solutions were prepared fresh: BPSoln1 (3% w/v bovine serum albumin/BSA + 0.05% v/v Triton-X in PBS) and BPSoln2 (5% BSA w/v + 0.1% v/v Triton-X in PBS). At selected time points during culture, encapsulated (“on-top”) cells were rinsed (2 × 5 min, PBS) and fixed (1 × 15 min) in 4% paraformaldehyde (PFA) in PBS. PFA was removed, hydrogels were washed (1 × 5 min PBS, 2 × 5 min BPSoln1), and the fixed cells were permeabilized and blocked (1 × 60 min, BPSoln2). After blocking, cells were incubated overnight at 4 °C with primary antibodies (Ki-67, Abcam, 1:100 dilution) in BPSoln2. The next day, hydrogels were rinsed (3 × 60 min, BPSoln1), incubated with secondary antibodies Alexa Fluor 488 goat-anti-mouse (ThermoFisher, 1:300 dilution) and F-actin (Sigma Aldrich, 1:250 dilution) in BPSoln2 overnight at 4 °C, and protected from light. Pre-conjugated AlexaFluor antibodies (AF488 Vimentin, BD Biosciences, 1:300 dilution; AF647 Ecadherin, BD Biosciences, 1:100 dilution) were incubated in the dark overnight at 4 °C in BPSoln2. On the final day of immunostaining, hydrogels were rinsed (3 × 45 min, BPSoln1) and incubated with DAPI (700 nM in PBS) for 1 h. Hydrogels were rinsed (3 × 30 min, PBS), stored at 4 °C in PBS, and protected from light, until imaging. Samples were imaged using a confocal microscope (Zeiss LSM 800). Three z-stacks (100 images per stack, 2 *μ*m spacing) were taken per hydrogel (n = 3), for a total of 9 images.

### β1 blocking

Antibody, AIIB2 (rat, Developmental Studies Hybridoma Bank, University of Iowa; deposited by C. H. Damsky), was used to block β1 integrin on the surface of T47D, and MDA-MB-231 cells were harvested for encapsulation experiments. Prior to encapsulation, cell aliquots were suspended in DMEM containing 100 *μ*g/ml AIIB2 antibody and incubated for 1 h at 37 °C. Aliquots were centrifuged (1200 rpm, 3 min), and the cell pellet was resuspended in the hydrogel precursor for encapsulation. Growth medium supplemented with 100 *μ*g/ml AIIB2 was replaced every 48–72 h during culture of β1-blocked cells.

### Imaging analysis and statistics

Z-stack images were initially processed in Volocity 3D imaging analysis software. Specifically, the shape and size of clusters and cells were determined by analysis of the cytoskeletal staining (F-actin). Clusters and cells were identified by finding objects in the red (F-actin) channel. Filters to close (No. = 4) and fill holes in the object were applied to improve the precision of volume measurements. A noise filter (medium) was added to smooth the surface of clusters and cells for more accurate surface area measurements. Additionally, touching objects were separated at a value of ∼500 000 *μ*m^3^ and objects touching the edges of the image or with volume less than 1000 *μ*m^3^ (debris) were excluded. The volume, surface area, and shape factor of each cell or cluster in each image was reported.

MATLAB was used to calculate averages and standard errors for the cluster/cell data from Volocity. To compare results for matrix density, β1 blocking, and viability experiments, student's t-tests were performed. One way ANOVA with Tukey's multiple comparisons was performed on data from the individual peptide, peptide mixture, and timepoint experiments.

### RNA isolation and RNA-Seq pipeline methods

Breast cancer cells (T47D, MDA-MB-231) were cultured for 3 days in GFOGER or IKVAV matrices. Hydrogel samples for each condition (MDA-MB-231 cells cultured in IKVAV or GFOGER and T47D cells cultured in IKVAV or GFOGER) were transferred to microcentrifuge tubes and degraded with collagenase (250 U/ml) for 20 min at 37 °C and 5% CO_2_. Once hydrogels were completely degraded (e.g., liquid solution that could be pipetted), cells were centrifuged (1200 RPM, 3 min), rinsed with 500 *μ*l of PBS to remove residual polymer and collagenase, followed by centrifugation, and then lysed with buffer for RNA isolation. Three degraded hydrogel samples were combined for each RNA replicate. Total RNA for three replicates was extracted using a Nucleospin miRNA kit (Takara). RNA concentrations were then quantified using a Qubit Fluorometer (Invitrogen), and RNA integrity was assessed using the AATI Fragment Analyzer (Advanced Analytical Technologies, Inc.). Samples with RQN (RNA quality number) ≥ 6 were used for this study.

Extracted RNA (3 replicates per condition) was sequenced by the Sequencing and Genotyping Center at the Delaware Biotechnology Institute. Briefly, indexed libraries were constructed from 100–500 ng of total RNA using the Universal Plus mRNA-seq Workflow Kit (Nugen) following the manufacturer's instructions. The molar quantity and quality of the libraries also were assessed by the Qubit and AATI Fragment Analyzer, respectively. The average library size was 400 bp. Sequencing was performed on an Illumina HiSeq2500 platform with 51-bp single-end reads to obtain ca. 25 million reads/sample. Raw sequence data and gene feature counts have been submitted to the NCBI under Gene Ontology Omnibus (GEO) project GSE121179.

Raw sequence data were analyzed by the Center for Bioinformatics and Computational Biology Core Facility at the University of Delaware using the existing RNA-Seq analysis pipeline.^85^ Briefly, initial quality control was performed using fastqc; reads were aligned to the human reference genome (Hg19) using Tophat2;^86^ mapping metrics were assessed using RseQC;^87^ and gene/exon feature counts were calculated using HTseq.^88^ Note that during these analyses, one sample (a replicate of MDA-MB-231 in IKVAV matrix) was a significant outlier and was removed from differential expression analysis, as its significantly lower read mapping percentage and other quality characteristics indicated an RNA quality or other technical issue during library preparation.

### Bioinformatics analyses

A heatmap of the counts per million (cpm) for each of the 4 conditions at day 3 in culture was generated with the R function heatmap.2 from gplots package version 3.0.1.^89^ The cpms for the replicates of each sample were averaged, and the top 50 genes with the most variance across the 4 conditions were selected. Each row of the heatmap was scaled to a mean of 0 and a standard deviation of 1 (z-score) and reordered via clustering shown on the left side.

Differential expression analysis was performed for each cell line (T47D or MDA-MB-231) at day 3 to compare the cell response with different synthetic matrix compositions (GFOGER, IKVAV) using EdgeR.^90,91^ Differential expression p-values were corrected using the false discovery rate (FDR) method. Genes with FDR-adjusted p values < 0.05 and fold change > 2 or <0.5 were considered significantly differentially expressed. There were a total of 1296 genes for MDA-MB-231 cells meeting these criteria and 33 genes for the T47D cells. A Venn diagram was generated to illustrate the number of genes differentially expressed for the two cell lines and their overlap.

DAVID [version 6.8 via the R package, RDAVIDWebService (version 1.16.0)^92^] was used to perform GO enrichment analysis for the 1296 differentially expressed MDA-MB-231 genes. Enrichment was tested for the GO Biological Process terms in GOTERM_BP_FAT. The 192 terms with Benjamini-Hochberg corrected p-values < 0.05 were considered significantly enriched. A tree map was generated from those enriched terms with the REVIGO^93^ web interface (http://revigo.irb.hr/) using default parameters.

### Cell cycle

Hydrogels were transferred to a microcentrifuge tube and degraded with (0.5 ml) 10 U/ml collagenase for 20 min at 37 °C. Cells were dissociated with Accutase for 20 min at 37 °C. Hydrogels and cells were pipetted every 5–10 min to ensure mixing for degradation and dissociation. After removal of solutions, centrifugation at 3000 RPM for 3 min, and removal of supernatant, cells were rinsed 1× with PBS (300 *μ*l) per sample. Again, the solution was removed, and cells were fixed with (300 *μ*l) 70% EtOH overnight at 4 °C. The next day, fixing solution was removed, and cells were re-suspended with 1 *μ*g/ml of Propidium Iodide (PI) and 5 *μ*g/ml RNAse. Samples were processed on a flow cytometer (Novocyte; Acea Biosciences, San Diego, CA), 10000 counts per sample, for PI analysis.

### Gene expression analysis using qRT-PCR

RNA was isolated from ER− MDA-MB-231 and ER+ T-47D in 3D cultures (6 wt. % GFOGER, 6 w% IKVAV) and in 2D culture on plates (growth control) on day 3, as described earlier for RNA-seq. An iTaq Universal SYBR Green One-Step kit was used according to the manufacturer's instructions (Bio Rad; Hercules, CA) for assessing gene expression by real-time quantitative reverse transcription PCR (qRT-PCR). 10 *μ*l qRT-PCR reactions were run on a CFX96 Touch Real Time PCR Detection System (Bio-Rad; Hercules, CA) to measure Ct values. Primers for genes of interest consisted of *GAPDH*, *MMP1*, *FN1*, and *ITGB1* (supplementary material Table S9). Each sample reaction included: 300 nM forward and reverse primers, 20–60 ng of RNA, 0.125 *μ*l of reverse transcriptase, 5 *μ*l of SYBR Green mix, and nuclease-free H_2_O for the remaining volume.

ΔCt values were calculated as the difference between the gene of interest and the housekeeping gene (*GAPDH*). ΔΔCt values were calculated as the difference between the ΔCt of the 3D culture and ΔCt of the growth control (2D culture), for each respective cell line. Fold change or relative expression, −ΔΔCt for each 3D culture, was plotted (log2), where directionality was correlated with up- or down-regulation. A two-sided Mann-Whitney test was used for determining statistical significance in the cell response to different 3D culture conditions. 3 biological replicates with 2 technical replicates were used for each culture condition.

## SUPPLEMENTARY MATERIAL

See supplementary material for additional data on monomer and hydrogel characterization, cell responses, statistical analyses, and bioinformatics analyses.
